# Dr. Henry Darwin Didama

**Published:** 1905-11

**Authors:** 


Dr. HENRY DARWIN DIDAMA, of Syracuse, died at his home October 4, 1905, aged 82 years. He graduated in medicine at Albany Medical College in 1846. He was professor of medicine and dean of the college of medicine in Syracuse University from 1873 until his death. He was president of the Medical Society of the State of New York in 1880. He was prominent in local, state, and national medical societies and a practitioner who bore the respect and esteem of his colleagues, patients, and fellow citizens.

